# Cheminformatic Analysis and Machine Learning Modeling to Investigate Androgen Receptor Antagonists to Combat Prostate Cancer

**DOI:** 10.1021/acsomega.2c07346

**Published:** 2023-02-13

**Authors:** Tianshi Yu, Chanin Nantasenamat, Supicha Kachenton, Nuttapat Anuwongcharoen, Theeraphon Piacham

**Affiliations:** † Center of Data Mining and Biomedical informatics, Faculty of Medical Technology, Mahidol University, Bangkok 10700, Thailand; ‡ Streamlit Open Source, Snowflake Inc., San Mateo, California 94402, United States; § Department of Clinical Microbiology and Applied Technology, Faculty of Medical Technology, Mahidol University, Bangkok 10700, Thailand

## Abstract

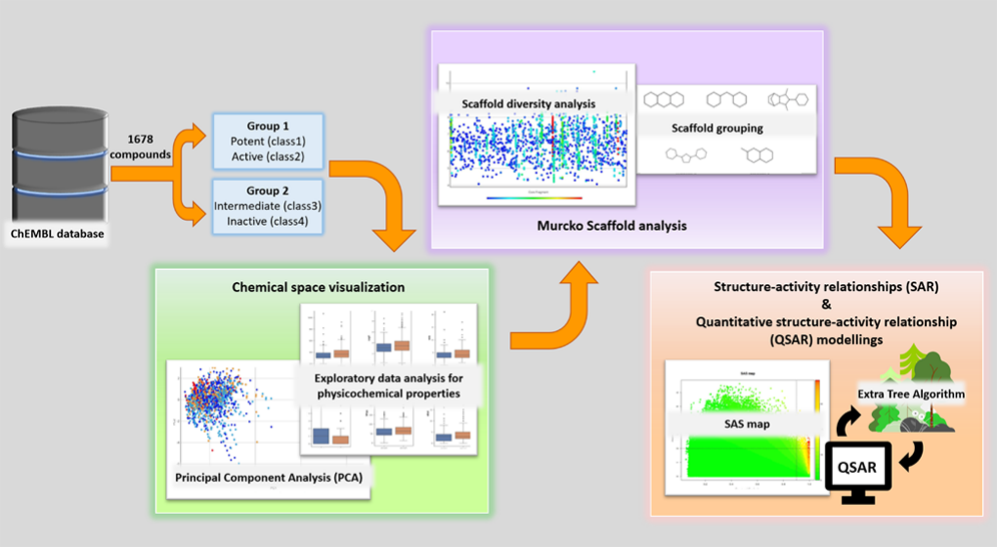

Prostate cancer (PCa) is a major leading cause of mortality of cancer among males. There have been numerous studies to develop antagonists against androgen receptor (AR), a crucial therapeutic target for PCa. This study is a systematic cheminformatic analysis and machine learning modeling to study the chemical space, scaffolds, structure–activity relationship, and landscape of human AR antagonists. There are 1678 molecules as final data sets. Chemical space visualization by physicochemical property visualization has demonstrated that molecules from the potent/active class generally have a mildly smaller molecular weight (MW), octanol–water partition coefficient (log *P*), number of hydrogen-bond acceptors (nHA), number of rotatable bonds (nRot), and topological polar surface area (TPSA) than molecules from intermediate/inactive class. The chemical space visualization in the principal component analysis (PCA) plot shows significant overlapping distributions between potent/active class molecules and intermediate/inactive class molecules; potent/active class molecules are intensively distributed, while intermediate/inactive class molecules are widely and sparsely distributed. Murcko scaffold analysis has shown low scaffold diversity in general, and scaffold diversity of potent/active class molecules is even lower than intermediate/inactive class molecules, indicating the necessity for developing molecules with novel scaffolds. Furthermore, scaffold visualization has identified 16 representative Murcko scaffolds. Among them, scaffolds 1, 2, 3, 4, 7, 8, 10, 11, 15, and 16 are highly favorable scaffolds due to their high scaffold enrichment factor values. Based on scaffold analysis, their local structure–activity relationships (SARs) were investigated and summarized. In addition, the global SAR landscape was explored by quantitative structure–activity relationship (QSAR) modelings and structure–activity landscape visualization. A QSAR classification model incorporating all of the 1678 molecules stands out as the best model from a total of 12 candidate models for AR antagonists (built on PubChem fingerprint, extra trees algorithm, accuracy for training set: 0.935, 10-fold cross-validation set: 0.735 and test set: 0.756). Deeper insights into the structure–activity landscape highlighted a total of seven significant activity cliff (AC) generators (ChEMBL molecule IDs: 160257, 418198, 4082265, 348918, 390728, 4080698, and 6530), which provide valuable SAR information for medicinal chemistry. The findings in this study provide new insights and guidelines for hit identification and lead optimization for the development of novel AR antagonists.

## 1 Introduction

Prostate cancer (PCa) is the second most prevalent among male cancer patients, as well as the fifth leading cause of mortality of cancer among males. There were 1,276,106 new cases causing 358,989 deaths in 2018^1^ and 1,414,259 new cases and 375,304 deaths in 2020.^2^ Majority of cases of PCa start with localized diseases. Localized diseases can be asymptomatic or mild symptoms, which can be tackled by active surveillance only. At this stage, the 5 year survival rate is nearly 100%. Then, a small percentage of cases proceed to locally advanced diseases, which are defined as the cancer tissues expanding beyond the prostate capsule but showing no lymph node spread or metastasis. For localized advanced cases, androgen deprivation therapy (ADT) through medication or surgery can slow down the progress of the disease. However, after the median of 24 months of ADT, treatment resistance is inevitable as demonstrated by a relapse of the biomarker: serum prostate specific antigen (PSA) levels. In the case of this, PCa progresses to castration-resistant prostate cancer (CRPC). And the 5 year survival rate is only 30%. There are a number of mechanisms underlying the pathogenesis and progression of PCa, and most are associated with androgen synthesis and androgen receptor (AR) signaling pathways.

**Figure 1:**
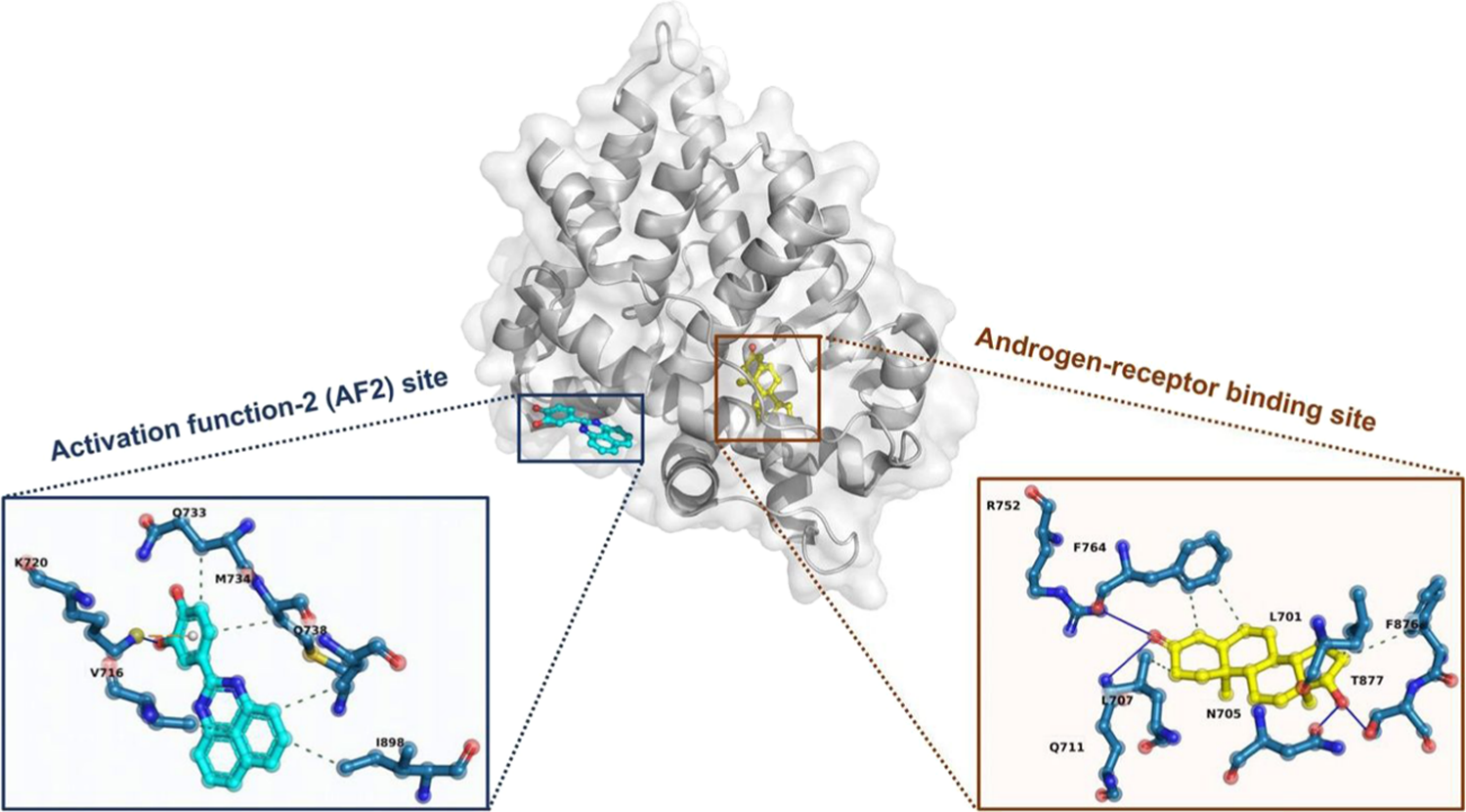
Three-dimensional (3D) crystal structure of ligand binding domain of androgen receptor (PDB ID: 2YHD).

Androgen receptor (AR) is a steroid receptor of the nuclear receptor superfamily. It functions as a transcription factor and regulates the development and growth of the prostate.^3^ The AR is bound to heat shock proteins (HSP90, HSP70) and molecular chaperones at the cytosol at its dormant state.^4,5^ When bound by an androgen molecule, it is released from the HSPs, transferred to the nucleus, and undergoes homologous dimerization to enter its active state as a transcriptional factor. AR is the pivotal regulator for PCa pathogenesis and progression. In most PCa cases, AR is overexpressed and is the driving force for the disease progression to CRPC. Therefore, it has become a highly significant therapeutic target for the drug discovery against PCa.^6,7^ AR has 919 amino acid residues and consists of three major domains: N-terminal domain (NTD) (residues 1–555), DNA binding domain (DBD) (residues 555–623), and the C-terminal ligand binding domain (LBD) (residues 665–919), which is connected to the DBD by a flexible hinge region (residues 623–665). All three domains are important for receptor function. The highly conserved DBD tethers the AR to promoter and enhancer regions of AR-regulated genes by direct DNA binding to allow the activation functions of the NTD and LBD, so that these genes can undergo transcription. Currently, there is no crystal structure for the full-length AR. However, the structures of both the DBD and LBD have been identified separately.^8,9^Figure 1 shows the crystal structure of LBD of AR (PDB ID: 2YHD). The LBD is composed of 11 α-helices and 4 short β strands that form two antiparallel β-sheets. With sandwich conformation, the 11 α-helices form a hydrophobic center to facilitate natural agonist binding.^3,10^ Various small molecules that act as AR agonists or AR antagonists exert their roles by binding to the ligand binding pocket in the LBD. LBD is currently the crucial target for the drug discovery of AR antagonists.

**Figure 2:**
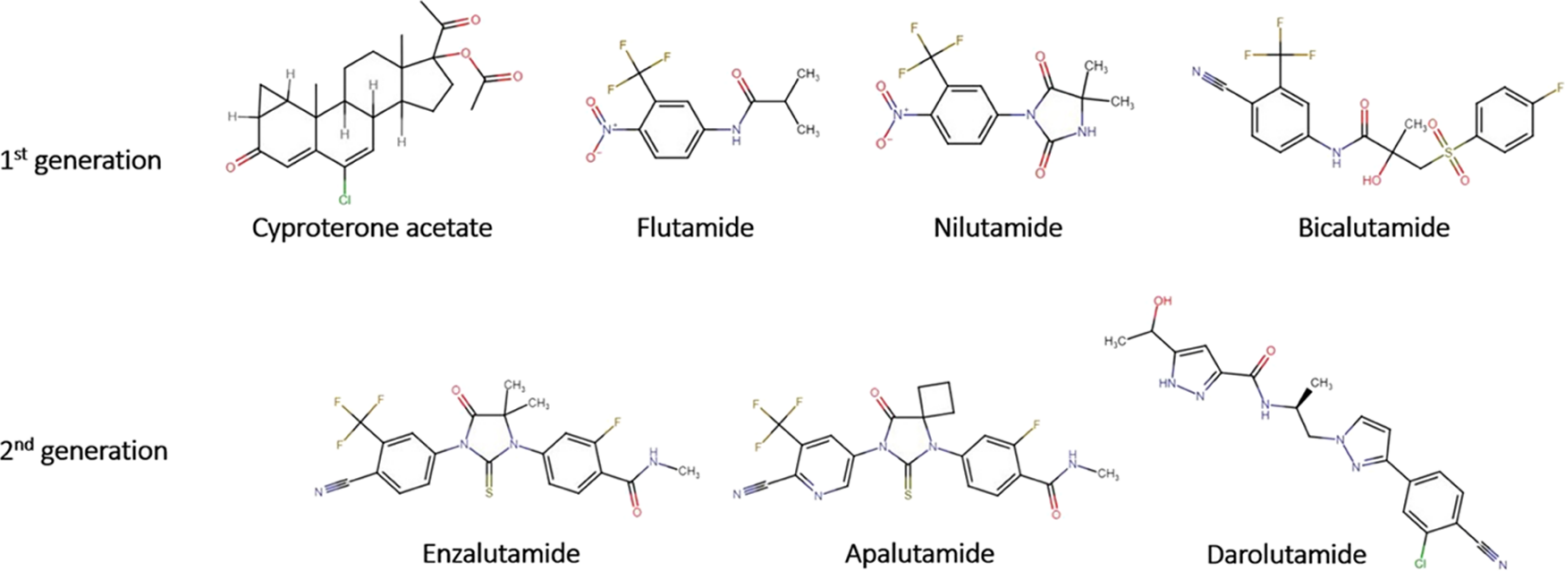
List of steroidal and two generations of nonsteroidal AR antagonists that have been approved by the FDA.

To treat PCa, especially CRPC, there have been numerous studies to develop AR antagonists. By chemistry, AR antagonists can be divided into two categories: steroidal antagonists and nonsteroidal antagonists. Cyproterone acetate is the representative steroidal AR antagonist,^11^ as shown in Figure 2. Due to the structural similarity to androgens, steroidal antagonists can interact with other steroid receptors leading to undesired side effects; they have been replaced by nonsteroidal antagonists, which demonstrate much better selectivity and safety profiles. There are two generations of nonsteroidal AR antagonists until now. First-generation drugs are represented by flutamide, nilutamide, and bicalutamide. They exert anti-AR functions through competitive inhibition of the LBD of AR.^3^ However, they have relatively weak binding affinities with AR, without capabilities of complete blockade of AR. In addition, they can induce mutations of LBD of AR along with treatment duration, leading to partial agonism to the mutated AR. Therefore, they are gradually replaced by the second-generation antagonists represented by enzalutamide, apalutamide, and darolutamide. In addition to the competitive inhibition of androgen-AR binding, second-generation antagonists also inhibit the AR translocation from cytoplasm to cell nucleus, the coactivator recruitment, and the AR-DNA binding.^12^ Although the second-generation antagonists display advantages over the first-generation antagonists, they have inevitably induced AR-resistant mutations that can render these drugs partial or mixed agonists for AR.^13^Figure 2 shows the first and second generations of AR antagonists. The mentioned challenges have posed urgent needs to develop novel antagonists against AR. Until now, based on the literature review, there are three trends with respect to the drug discovery of novel AR antagonists:^3,8^ first, the discovery of more potent molecules against LBD of AR by chemical modification of marketed drugs; second, due to the limited scaffold diversity of currently available antagonists against LBD of AR, more desirable chemical scaffolds need to be discovered and tried; last but not least, to try alternative domains of AR, such as NTD, as the binding site of novel antagonists.

Cheminformatics is a multidisciplinary field by utilization of computational and information technologies to find solutions for a wide range of problems in chemistry. It has achieved exponential progress in the era of machine learning and artificial intelligence (AI).^14^ In drug discovery, cheminformatics has long been applied to aid in the search for and optimization of new molecules. This paper performs systematic cheminformatic analysis and modeling to facilitate drug discovery of future AR antagonists. All of the data sets are compiled from the ChEMBL database. Cheminformatic analyses are performed to visualize the chemical space, investigate the distribution and patterns, identify representative Murcko scaffolds, and clarify structure–activity landscape. In addition, machine learning techniques are used to build quantitative structure–activity relationship (QSAR) classification models to better predict AR antagonistic bioactivities.

## 2 Materials and Methods

As a computational study, all of the biological activity data of the AR antagonists were compiled from ChEMBL database (ChEMBL version 31, target ID: 1871). Bioactivities labeled with IC_50_ are selected, and as a result, there are 3266 molecules obtained. Then comes the data cleansing process. First, 783 data sets that are without essential values (data sets that are without IC_50_ and pIC_50_ and those without “=”) are removed, so that 2483 data sets are left. Then, redundant data sets are removed by the ChEMBL molecule ID. In this step, a total of 805 duplicate data sets are removed. As a result, there are 1678 molecules left for the data set. Among them, there are 161 steroidal molecules and 1517 nonsteroidal molecules. Next, molecules with pIC_50_ ≥ 8 are labeled as potent, pIC_50_ between 7 and 8 as active, while 6 ≤ pIC_50_ < 7 as intermediate, and pIC_50_ < 6 as inactive. As a result, there are 122 potent, 432 active, 604 intermediate, and 520 inactive molecules.

For the cheminformatic analysis, all of the data sets are kept at original sizes. In the QSAR classification modeling stage, data sets are further balanced via the random oversampling technique. Namely, data sets randomly selected are duplicated within the potent, active, and inactive classes until their sizes are equal to the size of intermediate data (604 entries). The random state for oversampling is set to 42 to maintain reproducibility.

### 2.1 Chemical Space Visualization

#### 2.1.1 Chemical Space Visualization by Property Exploratory Data Analysis

In this step, all of the molecules are defined as group 1 (potent and active classes) and group 2 (intermediate and inactive classes). A total of six physicochemical properties are calculated, visualized, and compared between group 1 and group 2: molecular weight (MW), octanol–water partition coefficient (log *P*), number of hydrogen-bond acceptors (nHA), number of hydrogen-bond donors (nHD), number of rotatable bonds (nRot), and topological polar surface area (TPSA). DataWarrior^15^ (version 5.5.0) is used for the calculation of these properties.

In this section, the maximal, minimal, median, mean, skewness, and kurtosis are analyzed for the descriptors, and *p*-values between different groups are calculated to see if there’s any statistically significant difference. These values are obtained by programming in Pandas (version 1.4.0), jupyter notebook.

#### 2.1.2 Chemical Space Visualization by PCA

In this study, the six physicochemical properties are dimensionally reduced by principal component analysis (PCA). DataWarrior^15^ (version 5.5.0) is used for PCA.

### 2.2 Murcko Scaffold Analysis

#### 2.2.1 Murcko Scaffold Visualization

In this study, Murcko scaffolds and cyclic skeleton systems are obtained and compared by pIC_50_ levels, so that the distribution patterns of scaffolds can be identified and further analyzed. In addition, the frequency of skeletons and scaffolds is calculated and ranked. DataWarrior (version 5.5.0) is used for Murcko scaffold generation and visualization.

#### 2.2.2 Murcko Scaffold Diversity Analysis

Murcko scaffold diversity is calculated as the proportion of the number of Murcko scaffolds, number of singleton scaffolds (scaffold that possesses a single molecule), and number of Murcko skeletons to the total number of molecules.

#### 2.2.3 Scaffold Enrichment Factor (EF) Calculation

Scaffold enrichment factor (EF) is the ratio of the proportion of active molecules with a given scaffold to the proportion of active molecules in the entire data set.^16^ The molecular scaffolds with the higher EF are more desirable and vice versa.

### 2.3 Structure–Activity Landscape Visualization

In this study, the SAS map and SALI value are used to visualize the structure–activity landscape and identify activity cliffs (AC)s. In this study, Activity Landscape Plotter V.1, a webserver to generate SAS maps, is used.^17^ The threshold of structure and activity similarity are set to 0.9 and 2, respectively, which indicates that the activity cliff quadrant is defined as *X* > 0.9 and *Y* > 2. And the molecular fingerprints used for generating the SAS map consist of ECFP4, PubChem, and MACCS. Each set of fingerprints can generate the corresponding SAS maps and ACs, and the overlapping ACs are defined as consensus ACs.

### 2.4 Machine Learning-Based QSAR Modeling

**Figure 3:**
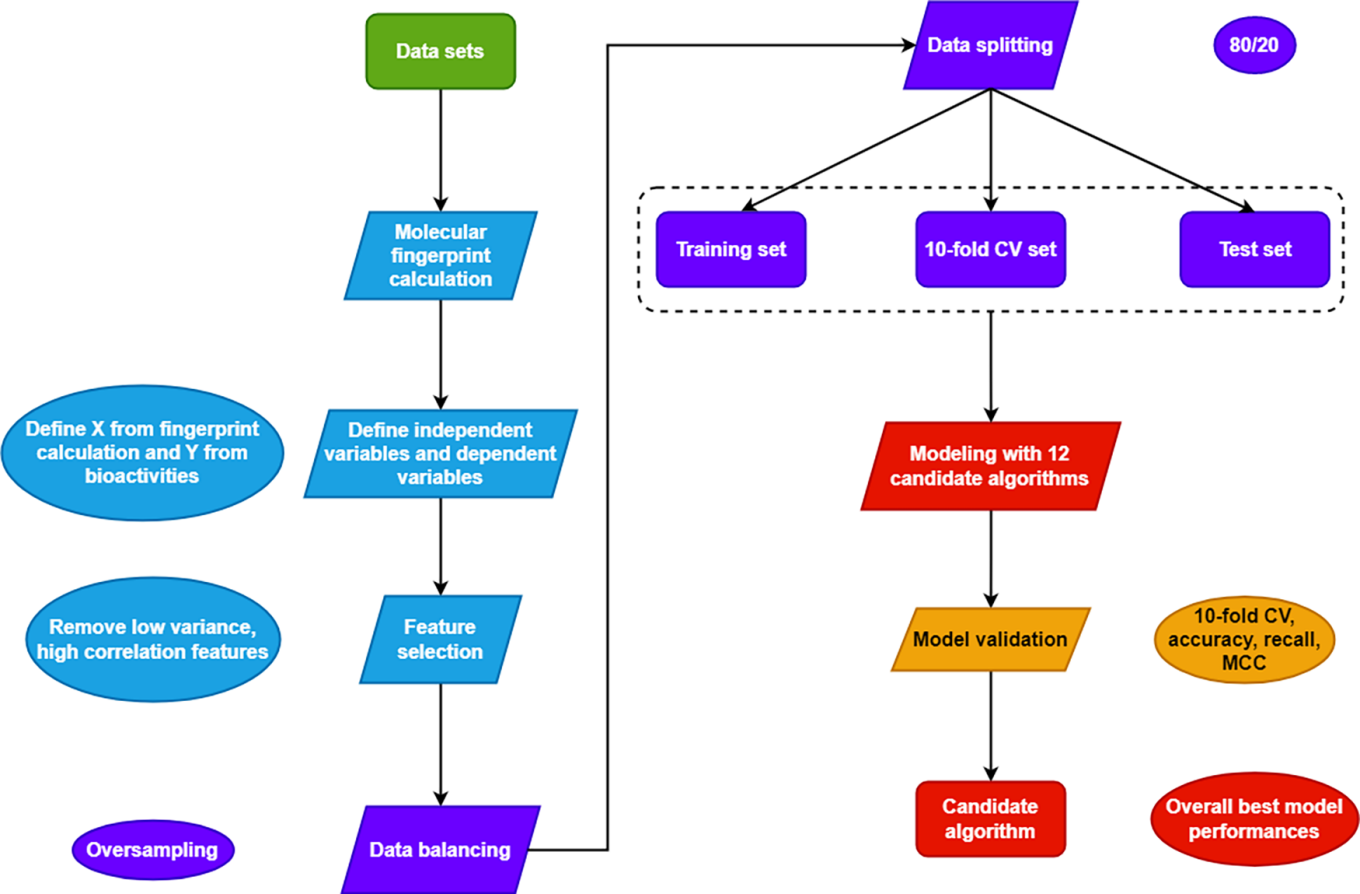
Schematic diagram for QSAR modeling in this study.

The schematic diagram for the modeling process is shown in Figure 3. All of the modeling processes are done using the python programming language, in Google Colab, facilitated by the Scikitlearn package (version 1.0.2).

#### 2.4.1 Molecular Fingerprints

PubChem fingerprints provided by the PaDEL package (PaDELpy-0.1.13) were used for modeling.^18^ The fingerprint set contains 881 binary representations of the chemical structural fragments used by PubChem. The parameter for the PaDEL package is set to detect aromaticity: true; standardize nitrogen: true; standardize tautomers: true; threads = 2; remove salt: true; log = true; fingerprints = true.

#### 2.4.2 Feature Selection

Features with variance lower than 0.1 and features demonstrating high correlation (>0.95) were removed. As a result, after feature selection of the 881 features, there are 223 left after the removal of low-variance features and 145 features were left after removing high correlation features.

#### 2.4.3 QSAR Model Construction

For all 12 models, the ratio of the training set and testing set is set to 80:20. Within the training set, a 10-fold cross-validation was performed to guarantee the robustness and reliability of the model. In addition, a one-vs-rest (OVR) strategy is employed for the multiclass classification. To get the best model, 12 representative classification algorithms^19−21^ have been used independently for model construction as shown in Table 1. Their performances are evaluated, compared, and the algorithm yielding the best performance will be taken.

**Table 1:** Machine Learning Algorithms for Modeling

algorithm	type	description of hyperparameter setting
decision tree (DT)	tree model	random state = 42
extra trees (ET)	ensemble learning	*n*_estimators = 500
		random state = 42
random forest (RF)	ensemble learning	max_features = 3
		*n*_estimators = 500
		random state = 42
		criterion = gini
gradient boost (GB)	ensemble learning	*n*_estimators = 500
		random state = 42
lightGBM (LGBM)	ensemble learning	*n*_estimators = 500
		random state = 42
extreme gradient boost (XGB)	ensemble learning	*n*_estimators = 500
		random state = 42
multilayer perceptron (MLP)	artificial neural network	hidden_layer_sizes = 100
		random state = 42
logistic regression (LR)	linear model	random state = 42
*K*-nearest neighbor (KNN)	nonparametric	default
support vector machine (SVM)	Kernel function	random state = 42
Naive-Bayes (NB)	Naive-Bayes	default
Gaussian process (GP)	nonparametric	random state = 42

#### 2.4.4 Performance Evaluation and Model Validation

The performance of the QSAR classification models was evaluated via three parameters: the accuracy (ac), the recall (re), and Matthew’s correlation coefficient (MCC). As a multiclass classification model, the recall is calculated by macroaverage. Let TP, TN, FP, and FN denote true positive, true negative, false positive, and false negative, respectively. The accuracy (eq 1), recall (eq 2), and MCC (eq 3) are defined as
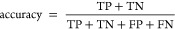
1
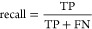
2
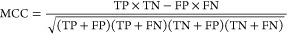
3

### 2.5 Structure–Activity Relationship Analysis by R-Group Decomposition

The Murcko scaffolds extracted previously are used for the automatic SAR in the DataWarrior platform (version 5.5.0). In this analysis, based on each specific scaffold, the R groups on different molecules are compared by pIC_50_ values to study their roles in determining bioactivities.

## 3 Results and Discussion

**Figure 4:**
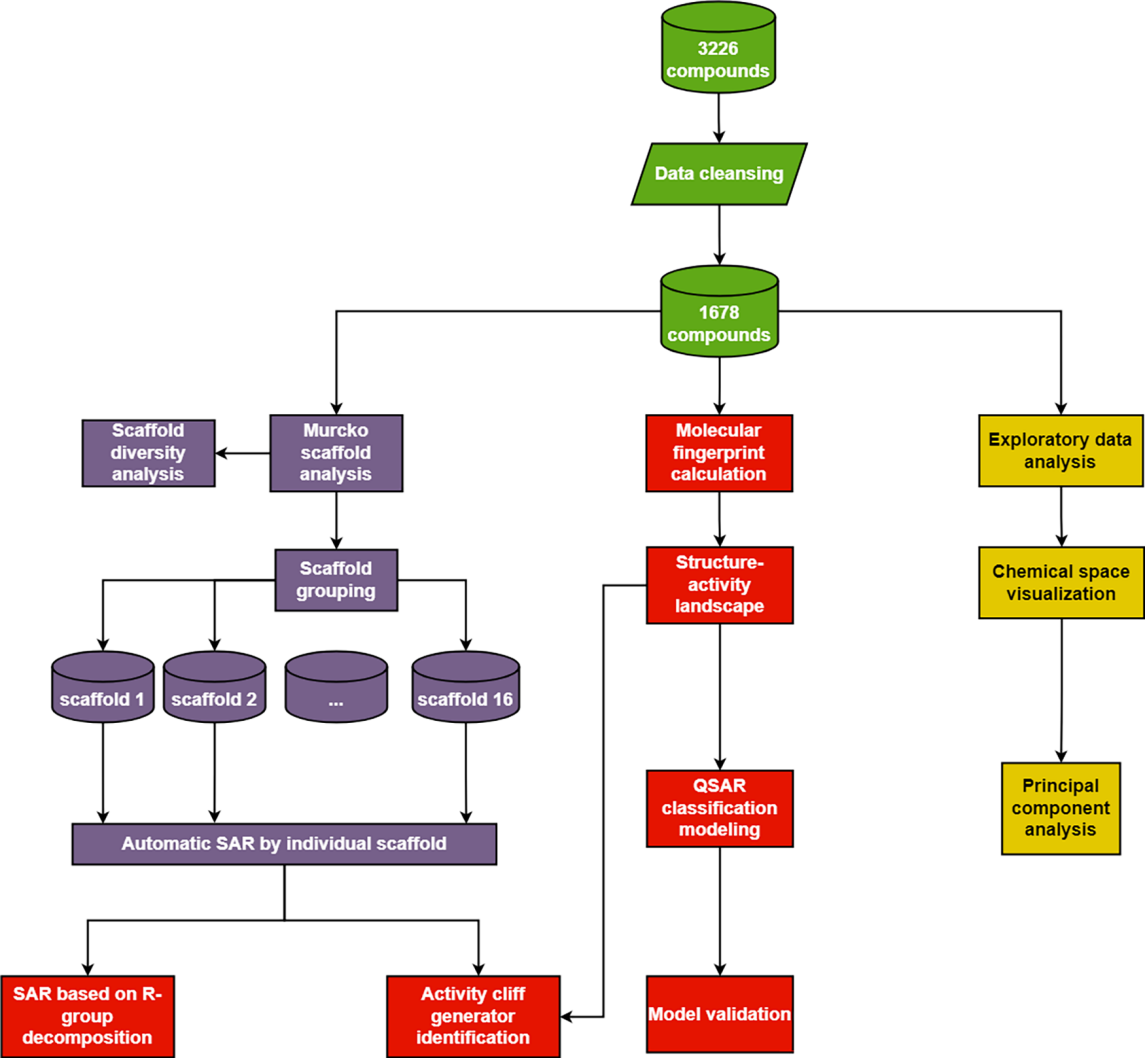
Overview of the workflow of the study.

As shown in Figure 4, there are a total of four colors: green for data compilation, yellow for exploratory data analysis and principal component analysis, both for chemical space visualization, purple for Murcko scaffold analysis, and red for structure–activity relationship studies. Furthermore, the workflow can be divided into five subsections: exploratory data analysis, chemical space visualization, scaffold analysis, structure–activity relationship, and identification of activity cliffs. For scaffold analysis and structure–activity landscape, including activity cliff generators, only the most essential figures and tables are demonstrated in this section, while the rest of them are in the Additional File.

### 3.1 Exploratory Data Analysis

**Figure 5:**
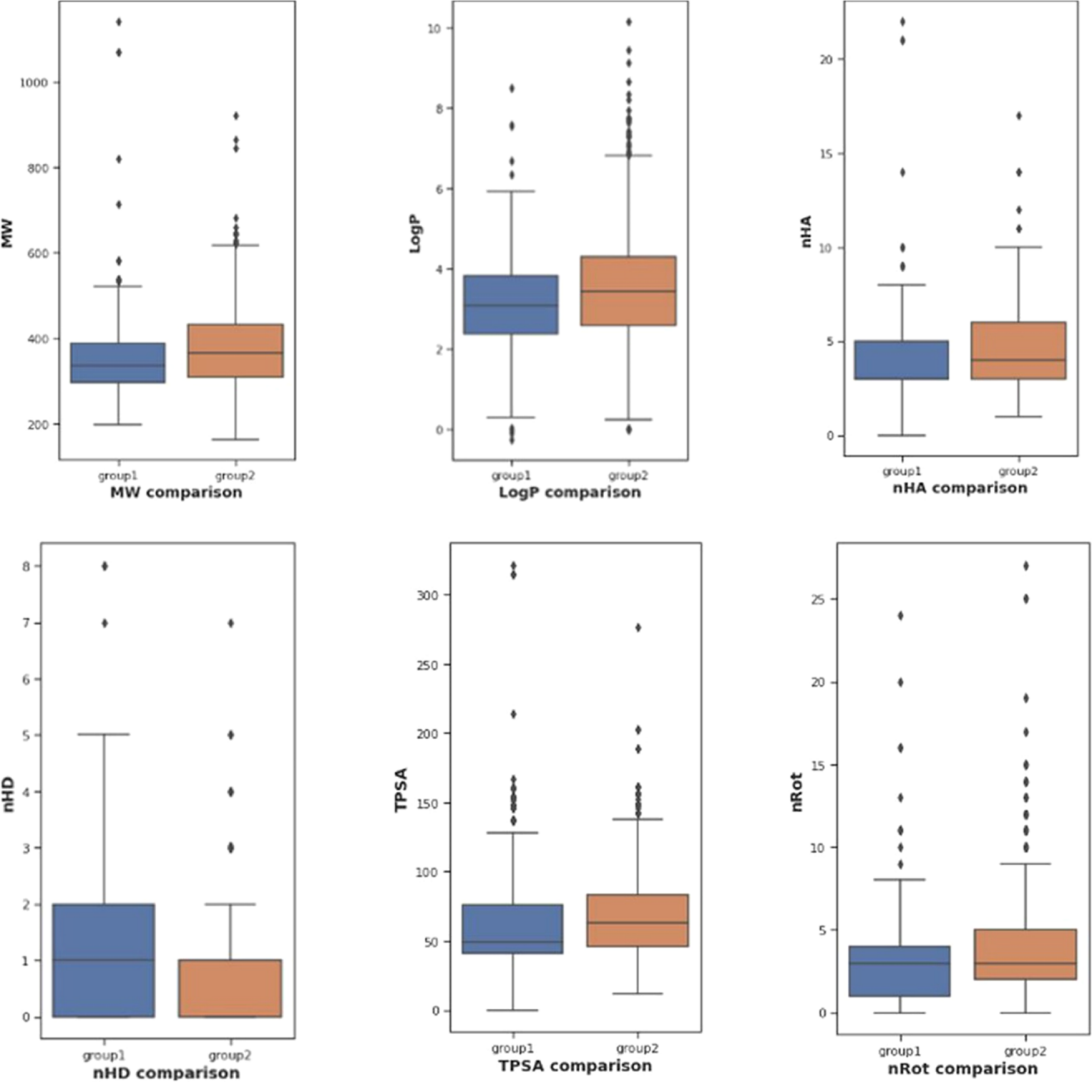
Exploratory analysis for six physicochemical properties.

Exploratory data analysis aims to explore the AR antagonists’ physicochemical property ranges, distributions, and patterns to get an overview of chemical space. It is the beginning of this study. As shown in Figure 5 and Table 2, all of the six physicochemical properties demonstrate nonparametric distribution patterns; the Mann–Whitney U test was performed to evaluate the statistical significance between group 1 and group 2. After the U test, all of the six properties have statistical significance (Table 2). Molecules from group 1 generally have mildly smaller MW, log *P*, nHA, nRot, and TPSA. The skew of group 1 has a much higher value in MW, nHA, and TPSA, which means that the distribution of these properties is more skewed. The kurtosis of group 1 has much higher values in MW, nHA, nHD, nRot, and TPSA, meaning that the distributions are highly peaked, heavily tailed, and leptokurtic, and the properties in group 1 are intensely distributed within a small region. It is important to note that exploratory data analysis by property distribution patterns is the preliminary visualization of chemical space. There are six properties or six dimensions to analyze the molecules. To get further insight into the chemical space, dimensionality reduction techniques are required to get more straightforward visualizations. Principal component analysis, or PCA, is an unsupervised machine learning approach used to reduce the dimensionality of large data sets by transforming large data sets into smaller data sets that still contain most of the information of the large set. In PCA, sets of correlated variables in a higher-dimensional space are combined to produce a set of variables in a lower-dimensional space.^19^

**Table 2:** Exploratory Data Analysis of Six Physicochemical Properties and Comparison between Bioactivity Classes^a^

	MW		log *P*		nHA		nHD		nRot		TPSA	
	group 1	group 2	group 1	group 2	group 1	group 2	group 1	group 2	group 1	group 2	group 1	group 2
*p*-value	2.147 × 10^–11^		1.031 × 10^–9^		2.230 × 10^–6^		0.015		1.282 × 10^–13^		2.041 × 10^–7^	
min	198.224	164.203	–0.25	–0.02	0	1	0	0	0	0	0	12.03
max	1140.08	921.001	8.499	10.158	22	17	8	7	24	27	321.12	276.88
median	336.353	366.499	3.082	3.425	3	4	1	1	3	3	49.005	63.21
mean	348.611	374.877	3.084	3.562	4.155	4.516	1.036	0.939	2.949	3.863	62.387	67.276
skew	3.358	0.966	0.265	0.67	2.99	0.866	2.157	1.031	2.993	2.338	2.873	1.057
kurtosis	23.006	2.612	1.841	1.038	17.9	1.745	13.559	2.578	17.904	11.127	15.319	2.89

a The *p*-value denotes the Mann–Whitney U test result.

### 3.2 Chemical Space Visualization by PCA

**Figure 6:**
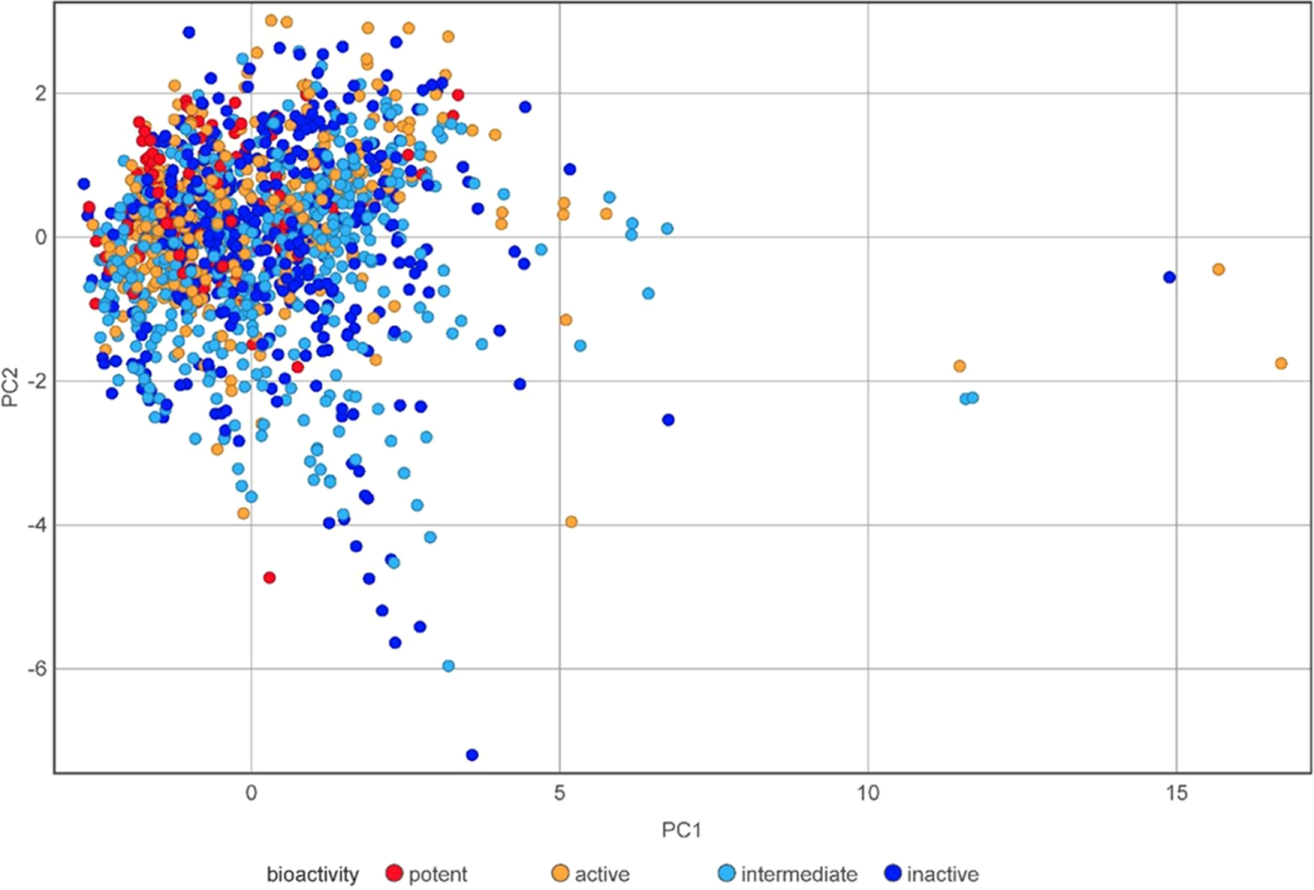
Chemical space visualization for AR antagonists by the PCA 2D plot using six physicochemical properties. To distinguish between different bioactivity classes, red and orange indicate potent and active classes, and light blue and navy blue indicate intermediate and inactive classes.

The PCA plot with six physicochemical properties has shown that mostly the potent and active class molecules are contained in chemical space from intermediate and inactive classes. Potent or active class molecules that are not contained show overlapping with intermediate, inactive classes (Figure 6). The potent class occupies the most concentrated area within the chemical space, followed by the active class. Intermediate and inactive class molecules cover the biggest area of the chemical space. The distributions and patterns in the PCA plot prove that potent and active molecules demonstrate lower diversity levels than intermediate, inactive molecules. Table 3 presents the eigenvalues of the six properties, which revealed that PC1 is primarily contributed by some nHA (0.514) and TPSA (0.512), followed by MW (0.471) and nRot (0.433). PC2 has the highest loadings by nHA (0.254) and TPSA (0.234), while log *P* (−0.814) is the most significant negative contributor. The third PC has the highest loading by nHA (0.186) and MW (0.147), and nHD (−0.965) is the most significant negative contributor.

**Table 3:** Eigenvalues of the Six Properties

property	PC1	PC2	PC3
MW	0.471	–0.332	0.147
log P	–0.064	–0.814	0.007
nHA	0.514	0.254	0.186
nHD	0.245	–0.012	–0.965
TPSA	0.512	0.234	0.081
nRot	0.433	–0.330	0.071
cumulated variance (%)	52.583	76.144	90.603

In Sections 3.1 and 3.2, through exploratory data analysis and PCA of the physicochemical properties, the goal of visualizing the chemical space of the AR antagonists is achieved. It is important to note that chemical space visualization is the overview of the molecules at the general level. To get more specific information, the molecules should be investigated in depth. Therefore, scaffold analysis as a powerful tool to analyze molecules is necessitated.

### 3.3 Scaffold Analysis

**Figure 7:**
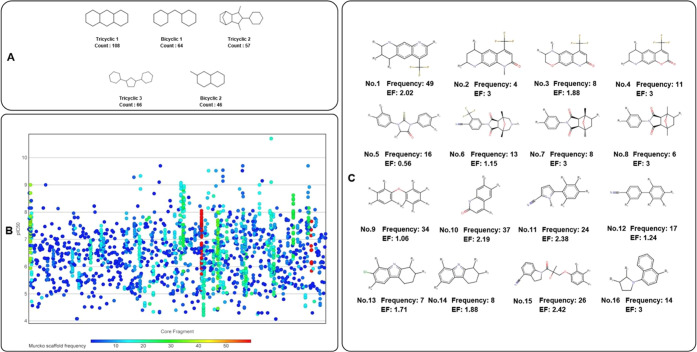
There are three subfigures based on scaffold analysis. (A) Top five frequent CSKs among nonsteroidal data sets. The counts are based on the total number of CSKs sharing the same ring systems and linkers, without distinguishing heteroatoms or aromatic/aliphatic bonds. (B) Murcko core fragment vs pIC_50_ plot. The *X* axis represents the core fragment based on Murcko scaffolds, and the *Y* axis represents the pIC_50_ values. The color of the dots means the Murcko scaffold frequency, the blue color for low frequency and the red color for high frequency. (C) Favorable Murcko scaffold-based core fragments for AR antagonists. In this section, the frequency of the core fragment within the complete data set and enrichment factor (EF) is described.

Scaffold analysis consists of three aspects: scaffold visualization, scaffold diversity analysis, and scaffold correlation with bioactivities. According to the visualization and frequency ranking, the top five most frequent CSKs are listed in Figure 7. Tricyclic scaffolds are the most significant, followed by bicyclic scaffolds.

Scaffold diversity is calculated as the proportion of the number of scaffolds to the total number of molecules (Table 4). The reason why Ns, Nss, and Ncsk of the four classes add up to exceed the complete data set is that among the four bioactivity classes, there are overlapping scaffolds shared by two or more bioactivity classes. The number in the complete data set is the add-up of all numbers truncating duplicate numbers.

**Table 4:** Murcko Scaffold Diversity Analysis

	number of molecules (*N*)	Murcko scaffold (Ns)	singleton Murcko scaffolds (Nss)	cyclic skeletons (Ncsk)	Ns/*N*	Nss/*N*	Ncsk/*N*	Ncsk/Ns
complete	1678	558	362	317	0.333	0.216	0.189	0.568
potent	122	47	27	33	0.385	0.221	0.270	0.702
active	432	166	95	105	0.384	0.220	0.243	0.633
intermediate	604	282	193	170	0.467	0.320	0.281	0.603
inactive	520	239	157	165	0.460	0.302	0.317	0.690

Murcko scaffold diversity analysis has demonstrated that scaffold diversity is low in general, and scaffold diversity of potent, active class molecules is lower than intermediate, inactive class molecules. Therefore, there is an urgent need to find more novel scaffolds for AR antagonists.

To correlate scaffolds with bioactivities, Murcko scaffolds are also plotted against bioactivity values to identify favorable scaffolds. Based on the plot, scaffold frequencies and EFs are calculated. There is a total of 16 representative scaffolds with either high frequencies or high EF values. Scaffolds 1–4 all belong to tricyclic 1 skeleton and they together have the highest frequency of molecules and high EF values, so that they are the most favorable scaffolds. In addition, scaffolds 10, 11, 14, and 15 all have a high frequency of molecules as well as high EF values. Scaffold 5 belongs to tricyclic 3 skeleton. In this study, scaffold 5 has EF < 1, which means that molecules with scaffold 5 have a lower proportion of potent/active classes than the overall average proportion. On the other hand, some representative second-generation AR antagonist drugs such as enzalutamide and apalutamide belong to scaffold 5. Therefore, the low EF value in scaffold 5 just represents the molecules compiled by this study. The role of scaffold 5 in the second-generation AR antagonist drugs has determined its significance in further lead optimization.

From this section, the structural in-depth information on AR antagonists has been extracted. To explore the activity information and to correlate with the structural information, SAR is explored using the R-group decomposition. From this procedure, local SAR information and valuable medicinal chemistry information are revealed for further chemical modifications. The SAR information is summarized in Table 5.

**Table 5:** Summary of 15 Series of SAR Information from the 16 Representative Scaffolds

scaffold	SAR	relevance with bioactivities
no. 1	hydroxyl group, nitrile group, halogens on R1;	indicating potent/active;
	amine group, sulfur-containing group on R1	indicating intermediate/inactive
no. 2	R1 and R2 positions are both aliphatic groups	no clear relevance
no. 3	3D spatial orientation of the carbon on the R2 position	largely affect
no. 4	R1 and R2 positions can be aliphatic groups or trifluoromethyl groups	no clear relevance
no. 5	halogen on the R1 position, isonitrile group on the R4 position, shorter chain on the R5 position;	counterproductive;
	longer aliphatic side chain with a peptide bond on the R5 position	beneficial
no. 6	ester groups on the R1 position	indicating potent/active
nos. 7 and 8	nitrile group on R3, trifluoromethyl group on R2	indicating potent/active
no. 9	nitrile group on R8 or R9;	indicating potent/active;
	fluoride or chloride substitution on R1 or R5	beneficial
no. 10	bis(2,2,2-trifluoroethyl)amine on R3	highly beneficial
no. 11	chloride or fluoride substitution on R2, R3, or R4;	indicating potent/active;
no. 12	long chain on R1	indicating intermediate/inactive
no. 13	chloride substitution on R1 or the presence of fluoride on R3	indicating intermediate/inactive
no. 14	nitrile group on R4;	indicating intermediate/inactive;
	halogen on R4	indicating potent/active
no. 15	nitrile group on either R1 or R3;	indicating potent/active;
	halogen substitutions on R1 or R3;	beneficial;
	oxygen-containing substitutions on R1 or R3	indicating intermediate/inactive
no. 16	nitrile group on R3	indicating potent/active

In Section 3.3, through comprehensive scaffold analysis, valuable information from the AR antagonist molecules was extracted. In Table 5, the SAR information is summarized, the structure–activity relationships and gained insights on further chemical modifications to optimize bioactivities were clarified. However, this section only provides SAR information of molecules with a specific scaffold, i.e., local SAR information. To gain the global SAR information of the AR antagonist molecules, we need to further utilize a machine learning approach to build QSAR models in the next section.

### 3.4 Structure–Activity Landscape Visualization and QSAR Modeling

**Figure 8:**
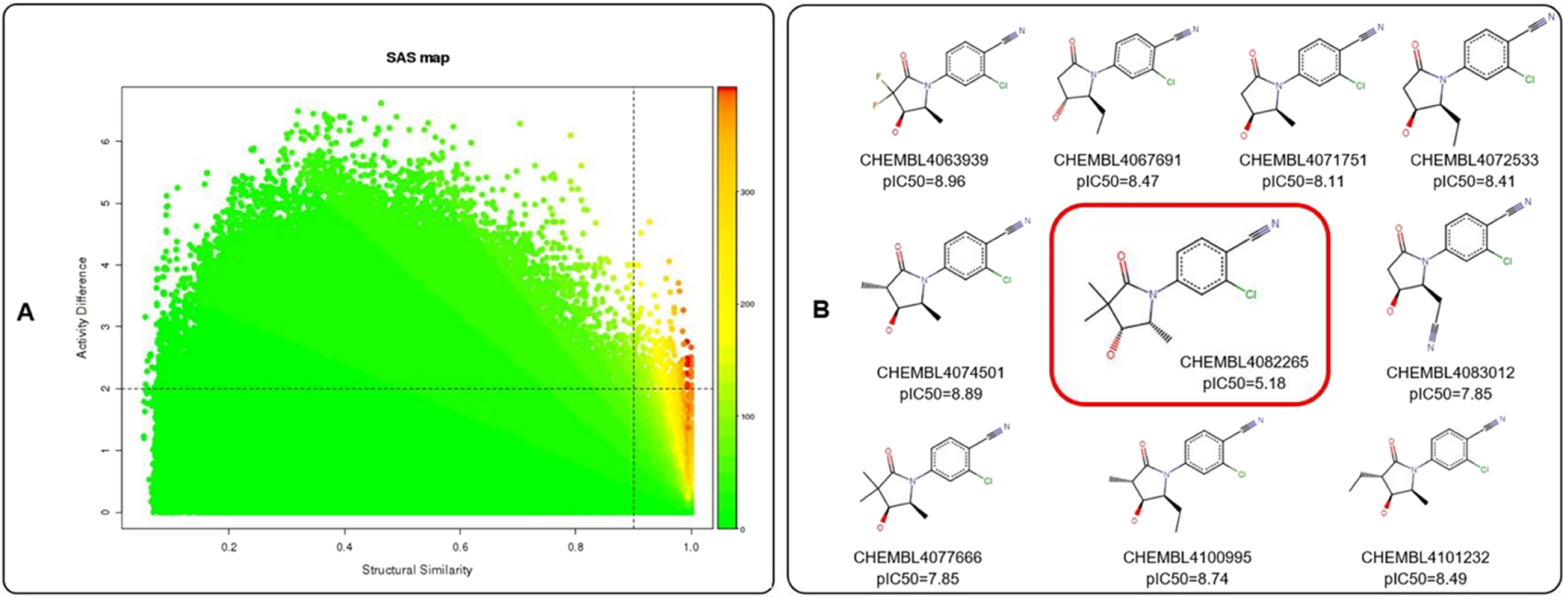
Panel (A) shows the SAS map of holistic data sets using PubChem fingerprint. The threshold for activity cliffs is set as *X* = 0.90 and *Y* = 2.0. The gradual change of color from green to red indicates the gradual increase of the SALI value. Panel (B) shows the representative AC generator (ChEMBL4082265) and the associated molecules that form ACs.

QSAR is short for the quantitative structure–activity relationship. It is a mathematical model to correlate structural data sets (PubChem fingerprint information in this study) of molecules with biological activities (bioactivities: potent, active, intermediate, and inactive classes in this study).^20^ Before executing QSAR modeling, a prerequisite is to evaluate the modelability of the data sets. In this study, structure–activity landscape visualization by the SAS map to evaluate modelability is performed (Figure 8A). SAS map is short for the structure–activity similarity map. This is a pairwise 2D plot of activity difference against structure similarity. The plot consists of two axes, *X*–*Y*, and four quadrants: smooth regions of the SAR space (lower right), rough region of activity cliffs (upper right), nondescript region (i.e., low structural similarity and low activity similarity) (lower left), and scaffold hopping region (low structural similarity but high activity similarity) (upper right). SALI value is short for the structure–activity landscape index. It is a pairwise measure between activity difference and structural difference for each pair of compounds. The higher SALI value, the higher potential of the pair of compounds forming ACs. In the SAS map, red color is used to highlight high SALI value pairs against green color, which indicates low SALI value. The SALI value is given in eq 4
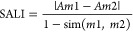
4Hereby, the letter *A* means activity, sim for similarity, and *m*1 and *m*2 are the abbreviations for molecule 1 and molecule 2, respectively. In this study, activity is represented by the pIC_50_ values of molecules, and similarity is represented by PubChem fingerprint similarity. In addition, the SALI value is utilized to quantitatively determine the existence of activity cliffs. SAS map has revealed that only a small percentage of pairs of compounds show a discontinuous structure–activity relationship. Based on the SAS map visualization and SALI values, it is concluded that the AR antagonist data sets are feasible to build the QSAR model.

Table 6 shows the summary of model performances with each of the 12 machine learning algorithms. Each of the 12 models incorporates all 1678 molecules, both steroidal and nonsteroidal. It is concluded that the extra trees (ET) algorithm provides the best model performance, with an accuracy of 0.935 in the training set, 0.735 in the 10-fold cross-validation set, and 0.756 in the testing set. The model can be used as a tool to predict the bioactivities of potential AR antagonists.

**Table 6:** Summary of Model Performance for the Complete Data Set of AR Antagonist Built Using the PubChem Fingerprint

	accuracy			recall			MCC		
	train	CV	test	train	CV	test	train	CV	test
DT	0.935	0.705	0.727	0.935	0.705	0.728	0.914	0.608	0.641
ET	0.935	0.735	0.756	0.935	0.735	0.757	0.914	0.648	0.678
RF	0.935	0.732	0.742	0.935	0.732	0.743	0.914	0.645	0.659
GB	0.897	0.713	0.736	0.897	0.713	0.737	0.863	0.618	0.651
LGBM	0.935	0.722	0.738	0.935	0.722	0.738	0.914	0.631	0.653
XGB	0.862	0.713	0.729	0.862	0.713	0.73	0.817	0.619	0.642
SVC	0.752	0.67	0.667	0.752	0.67	0.667	0.67	0.561	0.559
MLP	0.922	0.72	0.733	0.922	0.72	0.734	0.897	0.628	0.648
LR	0.71	0.619	0.655	0.71	0.619	0.652	0.614	0.493	0.542
KNN	0.764	0.66	0.663	0.764	0.66	0.663	0.687	0.551	0.557
NB	0.497	0.473	0.479	0.497	0.473	0.475	0.343	0.309	0.324
GP	0.921	0.712	0.733	0.921	0.712	0.735	0.896	0.618	0.649

In addition to QSAR modeling, according to the SAS map data set, there are 136 pairs of molecules that satisfy the threshold of ACs. Among the 136 pairs of molecules, there are seven significant AC generators (ChEMBL molecule IDs: 160257, 418198, 4082265, 348918, 390728, 4080698, and 6530) that can be seen as molecules with which they form a number of pairs of ACs. Activity cliff generators were identified as molecules highly frequent among ACs. The presence of ACs and AC generators is counterproductive to build machine learning models; however, they are of particular interest in medicinal chemistry to optimize lead compounds. The seven AC generators are listed in the Supporting Information. Among them, four molecules belong to tricyclic 1 Murcko skeleton. And each of the first- and second-ranked AC generators forms 28 and 13 ACs. There are lucrative molecules for further drug design.

The above are the results of this study and brief interpretations. A comprehensive understanding and interpretation of the outcomes of this study require discussing the current progress and knowledge gaps of AR antagonist drug discovery and comparing them with previous studies in computational drug discovery. In addition, the limitations of the study should be discussed, as well.

### 3.5 Current Progress and Knowledge Gaps of AR Antagonist Drug Discovery

AR signaling is the driving force for the growth and progression of PC. At its dormant state, AR forms a complex with heat shock proteins (HSP90, HSP70) and molecular chaperones at the cytosol. When bound by androgen or other agonists, it undergoes a conformational change that leads to its release from the HSPs. With the assistance of coactivators, AR is then transferred to the nucleus, recognizing androgen response elements (AREs) in a homologous dimerized form, entering its active state to regulate the expression of downstream genes. In addition to PC, AR signaling is associated with a series of hormone-related malignancies, such as breast cancer,^22^ ovarian cancer,^23^ pancreatic cancer,^24^ and even bladder cancer.^25^ Last but not least, AR signaling is a crucial pathway for human development and skeletomuscular integrity.^26^ Based on the role in various diseases or clinical implications, ligands of AR are generally divided into AR agonists, selective androgen receptor modulators (SARM),^27^ and AR antagonists. Table 7 presents the list of AR ligands by mechanisms of action.

**Table 7:** List of AR Ligands by Mechanisms of Action

	mechanism	application	examples
AR agonists	ligands that agonize the AR, as natural androgens or synthetic androgens, to upregulate the androgen signaling pathway		endogenous:
		delayed puberty;	testosterone
		hypogonadism;	dihydrotestosterone
		cryptorchidism;	synthetic:
		erectile dysfunctions	methyltestosterone
			nandrolone
SARM	selective androgen receptor modulators (SARMs) differentially bind to androgen receptors depending on the individual chemical structure. SARMs result in an anabolic state while avoiding many of the side effects of anabolic steroids such as inducing of PC	benign prostate hyperplasia;	ostarine
		cachexia;	VK5211
		Alzheimer’s disease;	GSK2881078
		osteoporosis;	
		muscular dystrophy;	
		breast cancer;	
		male contraception	
AR antagonists	ligands that antagonize the AR, by competitive inhibition or uncompetitive inhibition, to downregulate the androgen signaling pathway	prostate cancer treatment;transgender hormonal therapy	first generation:
			nilutamide
			flutamide
			bicalutamide
			second generation:
			enzalutamide
			apalutamide
			darolutamide
			emerging candidate:
			proxalutamide
			rezvilutamide
			EPI-506
			VPC-220010

There is a great deal of work associated with drug discovery for AR antagonists. Currently, until the year 2020, there have been two generations of AR antagonists approved for clinical implications. First-generation antagonists were dated back to the 1990s, i.e., flutamide, nilutamide, and bicalutamide. With the use of first-generation antagonists, patients eventually proceed to CRPC. Second-generation antagonists (enzalutamide, 2012; apalutamide, 2018; and darolutamide, 2019) have been approved since 2012, and they have much more potent binding affinities with AR than the first-generation antagonists.^3,12^ However, there are two challenges for second-generation antagonists: first, enzalutamide and apalutamide have off-target effects on the GABA-a receptors in the central nervous system and tend to induce seizures in a portion of patients. Second, like the first generation, they inevitably induce drug resistance in the long term. The mechanisms of resistance include point mutations (A587V, F876L, F877L, G684A, K631T, L595M, Q920R, R630Q, T576A, and T878A), AR alternative splicing leading to the absence of LBD on AR isoforms, and AR genetic amplification and enhanced transcription.^8^ Although there are some adaptations for second-generation antagonists as treatment regimens, the mechanisms that lead to resistance to first-generation antagonists inevitably render them useless. It is important to mention that second-generation AR antagonists share the same scaffold and the lack of scaffold diversity in antagonists could accelerate the proceedings of resistance. Based on the current challenges and situations, there are three recommendations for developing novel AR antagonists:

(1) Chemical optimization of the currently available molecules to obtain novel AR antagonists that can overcome or avoid resistance, largely targeting LBD;
(2) Using scaffold hopping and virtual screening to find novel AR antagonists of more diverse scaffolds;
(3) Focusing on alternative binding sites, such as NTD and DBD of AR, to overcome the drug resistance issue.

At the moment of this study, there are some novel AR antagonist candidates that have already proceeded to phase I/II clinical trials. Proxalutamide, also known as GT-0918, which shares the same scaffold with second-generation antagonists, has passed phase I clinical trial (clinical trial information: NCT02826772) with a 3-fold more potent binding affinity than enzalutamide and satisfactory tolerance.^28^ Phase II clinical trial (clinical trial information: NCT03899467) for mCRPC patients in the United States is expected to be complete by the end of 2022. It is revealed that GT-0918 as an orally available novel candidate drug acts not only by antagonizing AR but also by downregulating the lipogenesis through inhibiting the expression of ATP citrate lyase (ACL), acetyl CoA carboxylase (ACC), fatty acid synthase (FASN), and sterol regulatory element-binding protein-1 (SREBP-1).^28,29^ The coinhibition of AR signaling pathway and lipogenesis process confers GT-0918 more promising prospectus to overcome drug resistance and benefit patients’ survival. TRC-253, known as JNJ-63576253, is a novel, orally available novel candidate drug that has completed phase II/A clinical trial in Nov, 2020.^30^ As a lucrative pan-inhibitor against a series of mutant ARs as well as wild-type ARs, it is promising to be a novel resistance-overcome AR antagonist in the future. Rezvilutamide, known as SHR3680, is a novel candidate drug against AR that has completed phase II/A clinical trials (clinical trial information: NCT02691975) and is proven to be a potent AR antagonist with reduced CNS distribution, thereby decreasing the risk of inducing seizures in patients. Rezvilutamide is proven to be another promising candidate drug that is worth further clinical trials. TQB3720, a novel AR antagonist, is undergoing phase I clinical trial (clinical trial information: NCT04853498), and it is expected to be completed in 2023. BMS-641988 was used to be a promising candidate drug;^31^ however, phase I clinical trial (clinical trial information: NCT00644488; NCT00326586) has proven its potential to induce seizure in patients and further clinical trials have been discontinued. Among the emerging candidate drugs, proxalutamide, TRC-253, and rezvilutamide share the same scaffold structure with approved second-generation drugs, while BMS-641988 has a distinct scaffold structure. There should be more diverse scaffolds to facilitate more drug discoveries.

Currently, all of the clinically approved AR antagonists exert functions through the LBD of AR and the vast majority of candidate drugs are targeting LBD, as well. They are vulnerable to LBD point mutations and alternative splices. Apart from LBD, another domain on the AR, the NTD, is a lucrative novel target on AR, thanks to its essential role in AR transcription.^3^ Until now, there are several novel candidate drugs that antagonize AR functions via the NTD. They are the EPI-001, EPI-002 (EPI-506 as its prodrug), and VPC-220010.^32−34^ EPI series are natural products extracted from marine sponges. They are proved to be AR antagonists through NTD, as brand new target areas compared to other AR antagonists. Phase 1 clinical trial with EPI-506 has been terminated due to poor bioavailability and potency; however, safety profiles were satisfactory.^32^ Although the EPI-506 trial was terminated, it is a promising core structure for chemical modifications to find optimal NTD targeting AR antagonists. NTD as an intrinsically disordered domain on AR is not suitable for structure-based drug discovery for now; however, ligand-based optimization is highly recommended for further investigations. DBD is another domain on the AR that could be an alternative binding domain. DBD consists of two α-helices: P-box for recognition that binds transcription factor and D-box for AR dimerization. However, due to the fact that both the P-box and D-box motifs are highly conserved between steroid receptors, drug discovery for AR targeting of this domain can easily trigger off-target effects on other steroid receptors, such as progesterone receptor (PR) and glucocorticoid receptor (GR). Although challenging, there is some progress in DBD targeting AR antagonists, such as the discovery of pyrvinium, an anti-pinworm infection drug repurposed for AR antagonism, as a noncompetitive antagonist via DBD. As expected, pyrvinium demonstrated various antagonisms for PR and GR. Apart from this molecule, there are some other studies on the binding site, but none of them have proceeded to clinical trials for now.^35−37^

### 3.6 Comparisons with Previous Studies Using Cheminformatic or Computational Methods

There have been only a few previous studies using cheminformatic or computational methods to identify novel AR antagonists. The group of Hao et al. performed systemic cheminformatic analysis for AR agonists and antagonists using data sets from PubChem.^38^ And their study covered both AR agonists and antagonists. The group of Ban et al. has worked on computational drug discovery for AR antagonists using a structure-based drug discovery approach.^39^ The group of Paul et al. has developed a model that can predict the response of previously unreported AR mutants to current treatment pipelines for PCa,^40^ with an accuracy of 90%. Their model enables the prediction of the response of AR mutants by various ligands to see whether they act as agonists or antagonists. Their model has already been validated by external experimental evaluation. Apart from that, there are a number of studies focusing on QSAR/machine learning modelings of AR agonists/antagonists.^41−44^ Among the previous machine learning modeling studies, the project of CoMPARA, namely, the collaborative modeling project for androgen receptor activity, initiated by the U.S. Environmental Protection Agency (EPA) to explore endocrine-disrupting chemicals (EDC)s, is the most prominent project with large amounts of high-quality data sets of various origins, rigorous consensus model based on a total of 91 externally submitted models, and reliable model performance of 80% accuracy.^42^ However, the CoMPARA project is focused on evaluating environmental chemicals and toxicants that may play the roles of EDCs and is not applicable to drug discovery. In comparison with previous similar studies, this study uses data sets from the ChEMBL database, using a ligand-based drug discovery approach and cheminformatics, mainly by scaffold analysis, SAR, and QSAR modeling.

### 3.7 Limitations of This Study

As a computational study, all of the data sets of this study are compiled from the ChEMBL database, and they are retrospective records originating from various years and backgrounds for the bioactivities. Some previous and ongoing assays and experiments are not recorded in the ChEMBL database, so that there could be a lack of some valuable data sets, for example, additional valuable scaffolds, which is the main limitation of the study.

## 4 Conclusions

**Figure 9:**
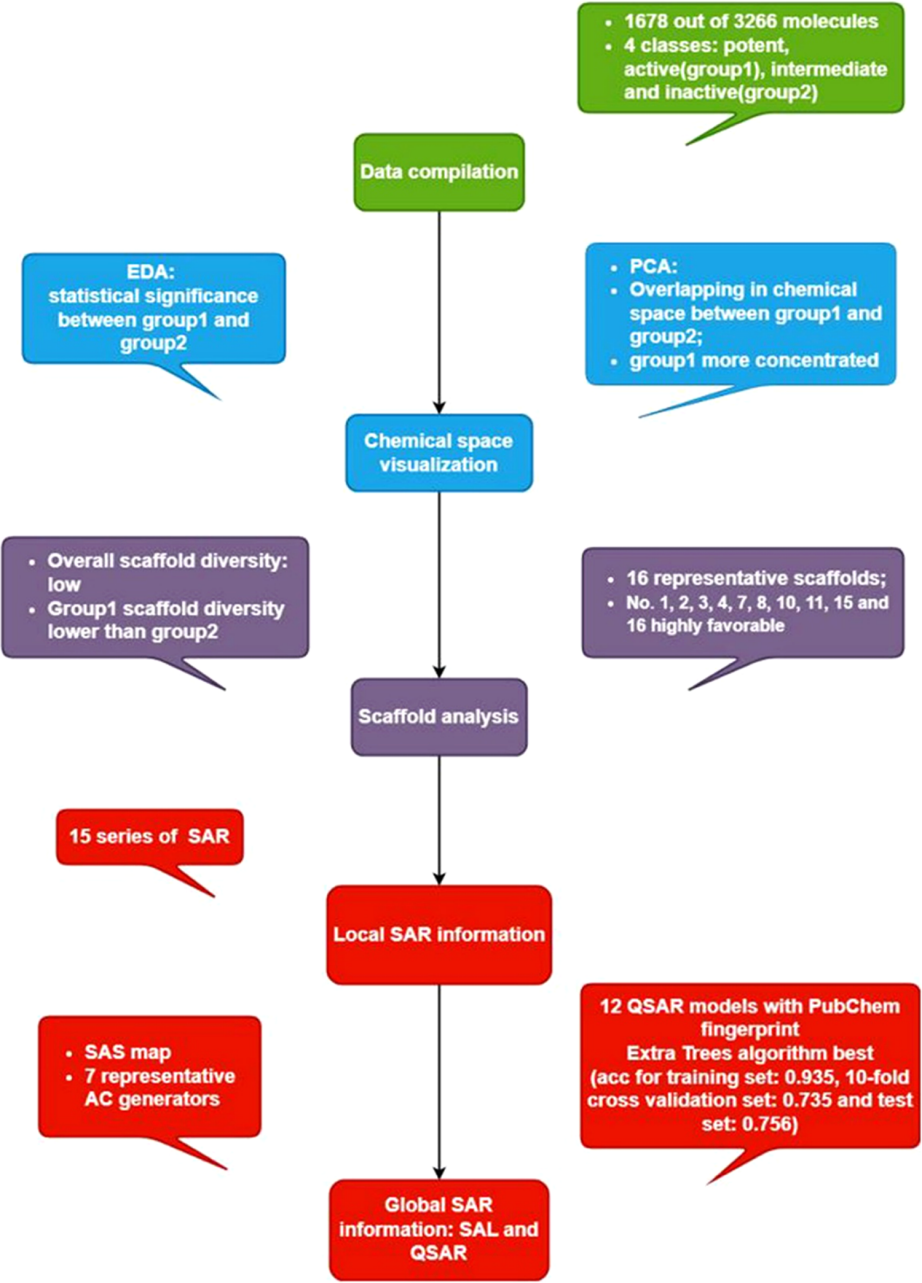
Graphical summary of the study.

Androgen receptor signaling is the driving force for the growth and progression of prostate cancer. Until now, there has been a great deal of work associated with drug discovery for novel AR antagonists. This study is a systematic cheminformatic analysis and machine learning modeling to visualize the chemical space, analyze the Murcko scaffold, and investigate the structure–activity relationship and landscape of human androgen receptor antagonists from the ChEMBL database. The graphical summary of this study is shown in Figure 9. At the data compilation procedure, 1678 out of 3266 data sets are extracted as the final input data sets. Based on bioactivity levels, the 1678 data sets are categorized into potent, active class (group 1) and intermediate and inactive (group 2) classes. To get an overview of the chemical space, EDA was executed and indicated statistical significance between group 1 and group 2 molecules in the physicochemical properties selected. Further visualization by PCA has demonstrated overlapping between group 1 and group 2 molecules, and group 1 molecules are more concentrated, almost contained by group 2, while group 2 molecules are widely, sparsely distributed. A deeper investigation of the molecules via scaffold analysis has identified 16 representative Murcko scaffolds. Among them, scaffolds 1, 2, 3, 4, 7, 8, 10, 11, 15, and 16 are highly favorable. And scaffold diversity analysis has further proved that scaffold diversity is low in general, and scaffold diversity of group 1 molecules is lower than group 2 molecules, indicating an urgent need to find more novel scaffolds for androgen receptor antagonists. There are 16 favorable Murcko scaffold-based core fragments extracted from scaffold analysis. Based on each of the core fragments, there are 15 series of structure–activity relationships, i.e., local (scaffold-based) structure–activity relationships clarified. Furthermore, to investigate the global structure–activity relationship, a total of 12 QSAR classification models using PubChem fingerprints are established for androgen receptor antagonists and the best one (with extra trees algorithm) provides accuracy for the training set: 0.935, 10-fold cross-validation set: 0.735 and test set: 0.756. Deeper insights into the structure–activity landscape highlighted seven significant activity cliff generators (ChEMBL molecule IDs: 160257, 418198, 4082265, 348918, 390728, 4080698, and 6530). These activity cliff generators provide practical information regarding further chemical modification and optimization. Findings in this study provide researchers with more specific insights into the property distributions, patterns, and scaffold structural information; furthermore, SAR, QSAR modelings, and AC generators can guide further drug discovery of novel AR antagonists to combat prostate cancer.

## Supporting Information Available

Additional file: representative pairs of activity cliff (PDF)

## References

[ref1] Rawla P. Epidemiology of Prostate Cancer. World J. Oncol. 2019, 10, 63–89. 10.14740/wjon1191. 31068988 PMC6497009

[ref2] Wang L.; Lu B.; He M.; Wang Y.; Wang Z.; Du L. Prostate Cancer Incidence and Mortality: Global Status and Temporal Trends in 89 Countries From 2000 to 2019. Front. Public Health 2022, 10, 811044 10.3389/fpubh.2022.811044. 35252092 PMC8888523

[ref3] Li D.; Zhou W.; Pang J.; Tang Q.; Zhong B.; Shen C.; Xiao L.; Hou T. A Magic Drug Target: Androgen Receptor. In Med. Res. Rev.; John Wiley and Sons Inc., 2019; pp 1485–1514. 10.1002/med.21558 30569509

[ref4] Stope M. B.; Schubert T.; Staar D.; Rönnau C.; Streitbörger A.; Kroeger N.; Kubisch C.; Zimmermann U.; Walther R.; Burchardt M. Effect of the Heat Shock Protein HSP27 on Androgen Receptor Expression and Function in Prostate Cancer Cells. World J. Urol. 2012, 30, 327–331. 10.1007/s00345-012-0843-z. 22362414

[ref5] J M B C T D L; Pnas J.; Performed Research. Targeting the Regulation of Androgen Receptor Signaling by the Heat Shock Protein 90 Cochaperone FKBP52 in Prostate Cancer Cells 2011; Vol. 108. 10.1073/pnas.1105160108 PMC3141981 21730179

[ref6] Fujita K.; Nonomura N. Role of Androgen Receptor in Prostate Cancer: A Review. World J. Mens Health 2019, 37, 288–295. 10.5534/wjmh.180040. 30209899 PMC6704300

[ref7] Robinson D.; van Allen E. M.; Wu Y. M.; Schultz N.; Lonigro R. J.; Mosquera J. M.; Montgomery B.; Taplin M. E.; Pritchard C. C.; Attard G.; Beltran H.; Abida W.; Bradley R. K.; Vinson J.; Cao X.; Vats P.; Kunju L. P.; Hussain M.; Feng F. Y.; Tomlins S. A.; Cooney K. A.; Smith D. C.; Brennan C.; Siddiqui J.; Mehra R.; Chen Y.; Rathkopf D. E.; Morris M. J.; Solomon S. B.; Durack J. C.; Reuter V. E.; Gopalan A.; Gao J.; Loda M.; Lis R. T.; Bowden M.; Balk S. P.; Gaviola G.; Sougnez C.; Gupta M.; Yu E. Y.; Mostaghel E. A.; Cheng H. H.; Mulcahy H.; True L. D.; Plymate S. R.; Dvinge H.; Ferraldeschi R.; Flohr P.; Miranda S.; Zafeiriou Z.; Tunariu N.; Mateo J.; Perez-Lopez R.; Demichelis F.; Robinson B. D.; Schiffman M.; Nanus D. M.; Tagawa S. T.; Sigaras A.; Eng K. W.; Elemento O.; Sboner A.; Heath E. I.; Scher H. I.; Pienta K. J.; Kantoff P.; de Bono J. S.; Rubin M. A.; Nelson P. S.; Garraway L. A.; Sawyers C. L.; Chinnaiyan A. M. Integrative Clinical Genomics of Advanced Prostate Cancer. Cell 2015, 161, 1215–1228. 10.1016/j.cell.2015.05.001. 26000489 PMC4484602

[ref8] Aurilio G.; Cimadamore A.; Mazzucchelli R.; Lopez-Beltran A.; Verri E.; Scarpelli M.; Massari F.; Cheng L.; Santoni M.; Montironi R. Androgen Receptor Signaling Pathway in Prostate Cancer: From Genetics to Clinical Applications. Cells 2020, 9, 2653 10.3390/cells9122653. 33321757 PMC7763510

[ref9] Callewaert L.; van Tilborgh N.; Claessens F. Interplay between Two Hormone-Independent Activation Domains in the Androgen Receptor. Cancer Res. 2006, 66, 543–553. 10.1158/0008-5472.CAN-05-2389. 16397271

[ref10] Matias P. M.; Donner P.; Coelho R.; Thomaz M.; Peixoto C.; Macedo S.; Otto N.; Joschko S.; Scholz P.; Wegg A.; Bäsler S.; Schäfer M.; Egner U.; Carrondo M. A. Structural Evidence for Ligand Specificity in the Binding Domain of the Human Androgen Receptor: Implications for Pathogenic Gene Mutations. J. Biol. Chem. 2000, 275, 26164–26171. 10.1074/jbc.M004571200. 10840043

[ref11] de Voogt H. J. The Position of Cyproterone Acetate (CPA), a Steroidal Anti-Androgen, in the Treatment of Prostate Cancer. Prostate 1992, 4, 91–95. 10.1002/pros.2990210514. 1533452

[ref12] Chen Y.; Zhou Q.; Hankey W.; Fang X.; Yuan F. Second Generation Androgen Receptor Antagonists and Challenges in Prostate Cancer Treatment. Cell Death Dis. 2022, 13, 632 10.1038/s41419-022-05084-1. 35864113 PMC9304354

[ref13] Rajaram P.; Rivera A.; Muthima K.; Olveda N.; Muchalski H.; Chen Q. H. Second-Generation Androgen Receptor Antagonists as Hormonal Therapeutics for Three Forms of Prostate Cancer. Molecules 2020, 25, 2448 10.3390/molecules25102448. 32456317 PMC7287767

[ref14] Miljković F.; Rodríguez-Pérez R.; Bajorath J. Impact of Artificial Intelligence on Compound Discovery, Design, and Synthesis. ACS Omega 2021, 6, 33293–33299. 10.1021/acsomega.1c05512. 34926881 PMC8674916

[ref15] Sander T.; Freyss J.; von Korff M.; Rufener C. DataWarrior: An Open-Source Program for Chemistry Aware Data Visualization and Analysis. J. Chem. Inf. Model. 2015, 55, 460–473. 10.1021/ci500588j. 25558886

[ref16] Manelfi C.; Gemei M.; Talarico C.; Cerchia C.; Fava A.; Lunghini F.; Beccari A. R. “Molecular Anatomy”: A New Multi-Dimensional Hierarchical Scaffold Analysis Tool. J Cheminf. 2021, 13, 54 10.1186/s13321-021-00526-y. PMC8299179 34301327

[ref17] González-Medina M.; Méndez-Lucio O.; Medina-Franco J. L. Activity Landscape Plotter: A Web-Based Application for the Analysis of Structure-Activity Relationships. J. Chem. Inf. Model. 2017, 57, 397–402. 10.1021/acs.jcim.6b00776. 28234475

[ref18] Yap C. W. PaDEL-Descriptor: An Open Source Software to Calculate Molecular Descriptors and Fingerprints. J. Comput. Chem. 2011, 32, 1466–1474. 10.1002/jcc.21707. 21425294

[ref19] Carracedo-Reboredo P.; Liñares-Blanco J.; Rodríguez-Fernández N.; Cedrón F.; Novoa F. J.; Carballal A.; Maojo V.; Pazos A.; Fernandez-Lozano C. A Review on Machine Learning Approaches and Trends in Drug Discovery. Comput. Struct. Biotechnol. J. 2021, 4538–4558. 10.1016/j.csbj.2021.08.011. 34471498 PMC8387781

[ref20] Nantasenamat C. Best Practices for Constructing Reproducible QSAR Models. In Ecotoxicological QSARs; Roy K., Ed.; Methods in Pharmacology and Toxicology; Humana Press Inc.: New York, NY, 2020; pp 55–75.

[ref21] Schaduangrat N.; Lampa S.; Simeon S.; Gleeson M. P.; Spjuth O.; Nantasenamat C. Towards Reproducible Computational Drug Discovery. J Cheminf. 2020, 12, 9 10.1186/s13321-020-0408-x. PMC6988305 33430992

[ref22] Chen M.; Yang Y.; Xu K.; Li L.; Huang J.; Qiu F. Androgen Receptor in Breast Cancer: From Bench to Bedside. Front. Endocrinol. 2020, 11, 573 10.3389/fendo.2020.00573. PMC7492540 32982970

[ref23] Mizushima T.; Miyamoto H. The Role of Androgen Receptor Signaling in Ovarian Cancer. Cells 2019, 8, 176 10.3390/cells8020176. 30791431 PMC6406955

[ref24] Kanda T.; Jiang X.; Yokosuka O. Androgen Receptor Signaling in Hepatocellular Carcinoma and Pancreatic Cancers. World J. Gastroenterol. 2014, 20, 9229–9236. 10.3748/wjg.v20.i28.9229. 25071315 PMC4110552

[ref25] Martínez-Rojo E.; Berumen L. C.; García-Alcocer G.; Escobar-Cabrera J. The Role of Androgens and Androgen Receptor in Human Bladder Cancer. Biomolecules 2021, 11, 594 10.3390/biom11040594. 33919565 PMC8072960

[ref26] Sinnesael M.; Claessens F.; Laurent M.; Dubois V.; Boonen S.; Deboel L.; Vanderschueren D. Androgen Receptor (AR) in Osteocytes Is Important for the Maintenance of Male Skeletal Integrity: Evidence from Targeted AR Disruption in Mouse Osteocytes. J. Bone Miner.Res. 2012, 27, 2535–2543. 10.1002/jbmr.1713. 22836391

[ref27] Solomon Z. J.; Mirabal J. R.; Mazur D. J.; Kohn T. P.; Lipshultz L. I.; Pastuszak A. W. Selective Androgen Receptor Modulators: Current Knowledge and Clinical Applications. Sex. Med. Rev. 2019, 7, 84–94. 10.1016/j.sxmr.2018.09.006. 30503797 PMC6326857

[ref28] Gu Y.; Xue M.; Wang Q.; Hong X.; Wang X.; Zhou F.; Sun J.; Peng Y.; Wang G. Novel Strategy of Proxalutamide for the Treatment of Prostate Cancer through Coordinated Blockade of Lipogenesis and Androgen Receptor Axis. Int. J. Mol. Sci. 2021, 22, 13222 10.3390/ijms222413222. 34948018 PMC8704202

[ref29] Zhou T.; Xu W.; Zhang W.; Sun Y.; Yan H.; Gao X.; Wang F.; Zhou Q.; Hou J.; Ren S.; Yang Q.; Yang B.; Xu C.; Zhou Q.; Wang M.; Chen C.; Sun Y. Preclinical Profile and Phase I Clinical Trial of a Novel Androgen Receptor Antagonist GT0918 in Castration-Resistant Prostate Cancer. Eur. J. Cancer 2020, 134, 29–40. 10.1016/j.ejca.2020.04.013. 32460179

[ref30] Rathkopf D. E.; Saleh M. N.; Tsai F. Y.-C.; Bilen M. A.; Rosen L. S.; Gottardis M.; Infante J. R.; Adams B. J.; Liu L.; Theuer C. P.; Freddo J. L.; Agarwal N. An Open Label Phase 1/2A Study to Evaluate the Safety, Pharmacokinetics, Pharmacodynamics, and Preliminary Efficacy of TRC253, an Androgen Receptor Antagonist, in Patients with Metastatic Castration-Resistant Prostate Cancer. J. Clin. Oncol. 2019, 37, e16542 10.1200/JCO.2019.37.15_suppl.e16542.

[ref31] Balog A.; Rampulla R.; Martin G. S.; Krystek S. R.; Attar R.; Dell-John J.; Dimarco J. D.; Fairfax D.; Gougoutas J.; Holst C. L.; Nation A.; Rizzo C.; Rossiter L. M.; Schweizer L.; Shan W.; Spergel S.; Spires T.; Cornelius G.; Gottardis M.; Trainor G.; Vite G. D.; Salvati M. E. Discovery of BMS-641988, a Novel Androgen Receptor Antagonist for the Treatment of Prostate Cancer. ACS Med. Chem. Lett. 2015, 6, 908–912. 10.1021/acsmedchemlett.5b00173. 26288692 PMC4538445

[ref32] Maurice-Dror C.; le Moigne R.; Vaishampayan U.; Montgomery R. B.; Gordon M. S.; Hong N. H.; DiMascio L.; Perabo F.; Chi K. N. A Phase 1 Study to Assess the Safety, Pharmacokinetics, and Anti-Tumor Activity of the Androgen Receptor n-Terminal Domain Inhibitor Epi-506 in Patients with Metastatic Castration-Resistant Prostate Cancer. Invest. New Drugs 2022, 40, 322–329. 10.1007/s10637-021-01202-6. 34843005

[ref33] Antonarakis E. S.; Chandhasin C.; Osbourne E.; Luo J.; Sadar M. D.; Perabo F. Targeting the N-Terminal Domain of the Androgen Receptor: A New Approach for the Treatment of Advanced Prostate Cancer. Oncologist 2016, 21, 1427–1435. 10.1634/theoncologist.2016-0161. 27628492 PMC5153341

[ref34] Yang Y. C.; Banuelos C. A.; Mawji N. R.; Wang J.; Kato M.; Haile S.; McEwan I. J.; Plymate S.; Sadar M. D. Targeting Androgen Receptor Activation Function-1 with EPI to Overcome Resistance Mechanisms in Castration-Resistant Prostate Cancer. Clin. Cancer Res. 2016, 22, 4466–4477. 10.1158/1078-0432.CCR-15-2901. 27140928 PMC5010454

[ref35] Radaeva M.; Ban F.; Zhang F.; Leblanc E.; Lallous N.; Rennie P. S.; Gleave M. E.; Cherkasov A. Development of Novel Inhibitors Targeting the D-Box of the Dna Binding Domain of Androgen Receptor. Int. J. Mol. Sci. 2021, 22, 2493 10.3390/ijms22052493. 33801338 PMC7958344

[ref36] Li H.; Ban F.; Dalal K.; Leblanc E.; Frewin K.; Ma D.; Adomat H.; Rennie P. S.; Cherkasov A. Discovery of Small-Molecule Inhibitors Selectively Targeting the DNA-Binding Domain of the Human Androgen Receptor. J. Med. Chem. 2014, 57, 6458–6467. 10.1021/jm500802j. 25062331

[ref37] Pal S. K.; Tew B. Y.; Lim M.; Stankavich B.; He M.; Pufall M.; Hu W.; Chen Y.; Jones J. O. Mechanistic Investigation of the Androgen Receptor DNA-Binding Domain Inhibitor Pyrvinium. ACS Omega 2019, 4, 2472–2481. 10.1021/acsomega.8b03205. 30873507 PMC6410682

[ref38] Hao M.; Bryant S. H.; Wang Y. Cheminformatics Analysis of the AR Agonist and Antagonist. J Cheminf. 2016, 8, 37 10.1186/s13321-016-0150-6. PMC4938998 27398098

[ref39] Ban F.; Dalal K.; LeBlanc E.; Morin H.; Rennie P. S.; Cherkasov A. Cheminformatics Driven Development of Novel Therapies for Drug Resistant Prostate Cancer. Mol. Inf. 2018, 37, 1800043 10.1002/minf.201800043. 29733509

[ref40] Paul N.; Carabet L. A.; Lallous N.; Yamazaki T.; Gleave M. E.; Rennie P. S.; Cherkasov A. Cheminformatics Modeling of Adverse Drug Responses by Clinically Relevant Mutants of Human Androgen Receptor. J. Chem. Inf. Model. 2016, 56, 2507–2516. 10.1021/acs.jcim.6b00400. 28024400

[ref41] Piir G.; Sild S.; Maran U. Binary and Multi-Class Classification for Androgen Receptor Agonists, Antagonists and Binders. Chemosphere 2021, 262, 128313 10.1016/j.chemosphere.2020.128313. 33182081

[ref42] Mansouri K.; Kleinstreuer N.; Abdelaziz A. M.; Alberga D.; Alves V. M.; Andersson P. L.; Andrade C. H.; Bai F.; Balabin I.; Ballabio D.; Benfenati E.; Bhhatarai B.; Boyer S.; Chen J.; Consonni V.; Farag S.; Fourches D.; García-Sosa A. T.; Gramatica P.; Grisoni F.; Grulke C. M.; Hong H.; Horvath D.; Hu X.; Huang R.; Jeliazkova N.; Li J.; Li X.; Liu H.; Manganelli S.; Mangiatordi G. F.; Maran U.; Marcou G.; Martin T.; Muratov E.; Nguyen D. T.; Nicolotti O.; Nikolov N. G.; Norinder U.; Papa E.; Petitjean M.; Piir G.; Pogodin P.; Poroikov V.; Qiao X.; Richard A. M.; Roncaglioni A.; Ruiz P.; Rupakheti C.; Sakkiah S.; Sangion A.; Schramm K. W.; Selvaraj C.; Shah I.; Sild S.; Sun L.; Taboureau O.; Tang Y.; Tetko Iv.; Todeschini R.; Tong W.; Trisciuzzi D.; Tropsha A.; van den Driessche G.; Varnek A.; Wang Z.; Wedebye E. B.; Williams A. J.; Xie H.; Zakharov A.; Zheng Z.; Judson R. S. Compara: Collaborative Modeling Project for Androgen Receptor Activity. Environ. Health Perspect. 2020, 128, 027002 10.1289/EHP5580. 32074470 PMC7064318

[ref43] Todorov M.; Mombelli E.; Aït-Aïssa S.; Mekenyan O. Androgen Receptor Binding Affinity: A QSAR Evaluation. SAR QSAR Environ. Res. 2011, 22, 265–291. 10.1080/1062936X.2011.569508. 21598194

[ref44] Wang Y.; Bai F.; Cao H.; Li J.; Liu H.; Gramatica P. A Combined Quantitative Structure-Activity Relationship Research of Quinolinone Derivatives as Androgen Receptor Antagonists. Comb. Chem. High Throughput Screening 2015, 18, 834–845. 10.2174/1386207318666150831125750. 26320943

