# Supplementary material for: Cheminformatic Analysis and Machine Learning Modeling to Investigate Androgen Receptor Antagonists to Combat Prostate Cancer
Source: ACS Omega. 2023 Feb 13;8(7):6729–42. doi: 10.1021/acsomega.2c07346 (PMC9948163; doi:10.1021/acsomega.2c07346)
Supplement: Supplementary file 1 — ao2c07346_si_001.pdf [file ao2c07346_si_001.pdf]

Cheminformatic analysis and machine learning modeling to investigate androgen receptor antagonists to combat prostate cancer

Tianshi Yu[1], Chanin Nantasenamat[2], Supicha Kachenton[3], Nuttapat Anuwongcharoen[1],

*Theeraphon Piacham[3]**

[1]Center of Data Mining and Biomedical informatics, Faculty of Medical Technology, Mahidol University, Bangkok, 10700, Thailand

[2] Streamlit Open Source, Snowflake Inc, Bozeman, Montana 59715, USA

[3]Department of Clinical Microbiology and Applied Technology, Faculty of Medical Technology, Mahidol University, Bangkok 10700, Thailand

*Correspondence: Theeraphon.pia@mahidol.ac.th

Table S1. List of seven representative AC generators. The ACs are extracted from SAS map using PubChem fingerprint. Only the most prevalent molecules are listed.

ChEMBL ID	2D structure	pIC_{50}	Pairs of ACs formed
160257	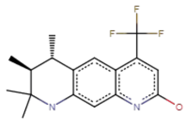	5.15	28
418198	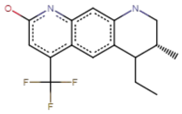	5.57	13
4082265	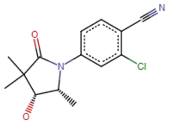	5.18	9
348918	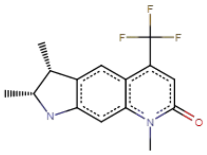	5.04	7
390728	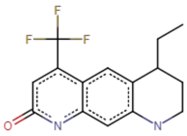	5.15	6

4080698	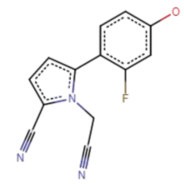	5.22	6
6530	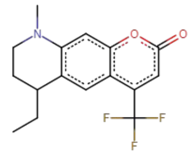	6.56	5
